# Cultivation-Independent and Cultivation-Dependent Analysis of Microbes in the Shallow-Sea Hydrothermal System Off Kueishantao Island, Taiwan: Unmasking Heterotrophic Bacterial Diversity and Functional Capacity

**DOI:** 10.3389/fmicb.2018.00279

**Published:** 2018-02-22

**Authors:** Kai Tang, Yao Zhang, Dan Lin, Yu Han, Chen-Tung A. Chen, Deli Wang, Yu-Shih Lin, Jia Sun, Qiang Zheng, Nianzhi Jiao

**Affiliations:** ^1^State Key Laboratory of Marine Environmental Science, Institute of Marine Microbes and Ecospheres, Xiamen University, Xiamen, China; ^2^Department of Oceanography, National Sun Yat-sen University, Kaohsiung, Taiwan

**Keywords:** shallow-sea hydrothermal system, metagenomics, genomics, heterotrophic bacteria, microbial community, *Rhodovulum*

## Abstract

Shallow-sea hydrothermal systems experience continuous fluctuations of physicochemical conditions due to seawater influx which generates variable habitats, affecting the phylogenetic composition and metabolic potential of microbial communities. Until recently, studies of submarine hydrothermal communities have focused primarily on chemolithoautotrophic organisms, however, there have been limited studies on heterotrophic bacteria. Here, fluorescence *in situ* hybridization, high throughput 16S rRNA gene amplicon sequencing, and functional metagenomes were used to assess microbial communities from the shallow-sea hydrothermal system off Kueishantao Island, Taiwan. The results showed that the shallow-sea hydrothermal system harbored not only autotrophic bacteria but abundant heterotrophic bacteria. The potential for marker genes sulfur oxidation and carbon fixation were detected in the metagenome datasets, suggesting a role for sulfur and carbon cycling in the shallow-sea hydrothermal system. Furthermore, the presence of diverse genes that encode transporters, glycoside hydrolases, and peptidase indicates the genetic potential for heterotrophic utilization of organic substrates. A total of 408 cultivable heterotrophic bacteria were isolated, in which the taxonomic families typically associated with oligotrophy, copiotrophy, and phototrophy were frequently found. The cultivation-independent and -dependent analyses performed herein show that Alphaproteobacteria and Gammaproteobacteria represent the dominant heterotrophs in the investigated shallow-sea hydrothermal system. Genomic and physiological characterization of a novel strain P5 obtained in this study, belonging to the genus *Rhodovulum* within Alphaproteobacteria, provides an example of heterotrophic bacteria with major functional capacity presented in the metagenome datasets. Collectively, in addition to autotrophic bacteria, the shallow-sea hydrothermal system also harbors many heterotrophic bacteria with versatile genetic potential to adapt to the unique environmental conditions.

## Introduction

Approximately 50–60 shallow-sea hydrothermal systems at depths of less than 200 m below sea level are currently known, occurring near active coastal or submarine volcanoes, with systems located along arcs, mid-ocean ridges, and in island arc-related environments and even in continental margins (Tarasov et al., 2005). While extensive microbial surveys of the deep-sea hydrothermal systems have been conducted since the first discovery of deep-sea hydrothermal vents nearly 40 years ago (Corliss et al., 1979), attention has also been paid to their shallow-sea counterparts, which are much easier to access and can often be explored via SCUBA diving (Tarasov et al., 2005). The biological data for nearly 30 shallow-sea hydrothermal vent ecosystems have been published (Tang, 2014). Previous surveys of 16S rRNA genes using tag pyrosequencing and clone libraries have revealed the composition of microbial communities in shallow-sea hydrothermal environments, including the shallow-sea hydrothermal systems located at Kueishantao Island off NE Taiwan (Zhang et al., 2012; Tang et al., 2013), Eolian Islands (Vulcano Island and Panarea Island) in Italy (Manini et al., 2008; Maugeri et al., 2009, 2010, 2013b), Ambitle Island (Meyer-Dombard et al., 2012), D. João de Castro Bank, Azores (Mohandass et al., 2012), Milos Island in Greece (Brinkhoff et al., 1999; Sievert et al., 1999, 2000; Bayraktarov et al., 2013; Giovannelli et al., 2013; Price et al., 2013), Taketomi Island in Japan (Hirayama et al., 2007), and Eyjafjordur in Iceland (Marteinsson et al., 2001). These investigations showed that there were frequently a high abundance of autotrophs within the classes Gammaproteobacteria and Campylobacteria (previously termed Epsilonproteobacteria) (Waite et al., 2017) in the shallow-sea systems.

The submarine hydrothermal systems harbor chemolithoautotrophic bacteria and archaea communities typically associated with locally reduced gasses sulfide (H_2_S), methane (CH_4_), and hydrogen (H_2_) (Grzymski et al., 2008; McDermott et al., 2015; Brazelton and Baross, 2010), which are considered to support primary productivity through carbon dioxide (CO_2_) fixation. The sulfur-reducing chemolithoautotrophs *Nautiliales*-like organisms within Campylobacteria and sulfide-oxidizing chemolithoautotrophs *Thiomicrospira*-like organisms within Gammaproteobacteria dominated and exhibited distinct zonation within the water columns of the shallow hydrothermal system off Kueishantao Island, Taiwan (Zhang et al., 2012; Tang et al., 2013). Chemolithoautotrophs *Nautiliales*-like and *Thiomicrospira*-like organisms are frequently found in the other shallow-sea systems (Hirayama et al., 2007), and were found in some deep-sea hydrothermal system as well (Scott et al., 2006; Campbell et al., 2009; Brazelton and Baross, 2010; Yamamoto and Takai, 2011). They might possess the reductive tricarboxylic acid cycle (rTCA) and the Calvin-Benson-Bassham (CBB) cycle for carbon fixation in response to the available energy source in the form of the oxidation of reduced sulfur compounds and hydrogen in the environment, which is possibly fueled by geochemical energy with hydrogen and reduced sulfur, respectively (Tang et al., 2013). Chemolithoautotrophic organisms were also active within the steep geochemical gradients of the shallow-sea hydrothermal sediments, which were possibly involved in sulfide oxidation and sulfate reduction (Bayraktarov et al., 2013; Giovannelli et al., 2013). The sediments often harbored abundant Campylobacteria (such as genus *Sulfurovum* that encompasses sulfur- and thiosulfate-oxidizers bacteria) similar to those seen in deep-sea vents, but also other proteobacterial lineages that are distinct from those of deep-sea vents (Giovannelli et al., 2013). These studies provided some indication of a potential biogeochemical function for chemolithoautotrophic organisms in the shallow-sea hydrothermal system with significant ramifications for sulfur and carbon cycles (Zhang et al., 2012; Bayraktarov et al., 2013; Giovannelli et al., 2013; Tang et al., 2013).

Inorganic carbon is the primary carbon source assimilated by autotrophic bacteria in submarine hydrothermal systems, but hydrothermal fluids can also carry elevated concentrations of dissolved and particulate organic matter (Bennett et al., 2011; Yang et al., 2012). In addition, fluids may cool down and change physicochemical fluctuating conditions by mixing rapidly with seawater (Yang et al., 2012). The enrichment of nutrients and the temperature and dissolved oxygen gradients in the mixed hydrothermal fluids and seawater might support active heterotrophic microbes. Recent studies have shown that deep-sea hydrothermal systems are inhabited by versatile heterotrophic Alphaproteobacteria and Gammaproteobacteria, which are significantly distinct from heterotrophic lineages common in the deep-sea environment, some of which have the potential for alkane degradation (Meier et al., 2016). A bacterium closely related to a human pathogenic *Vibrio* species was isolated from surrounding sulfide chimneys of a hydrothermal vent along the East Pacific Rise, and its genomic information provided new insights on how species adapt to the deep-sea environment (Hasan et al., 2015). Several studies have shown the presence of physiologically, metabolically, and phylogenetically diverse heterotrophic communities in shallow-sea hydrothermal systems (Giovannelli et al., 2013; Meyer-Dombard et al., 2013; Price et al., 2013), in which some seemed mostly to be involved in arsenic and iron redox cycling (Meyer-Dombard et al., 2013; Price et al., 2013). However, in marine hydrothermal systems, compared to chemolithoautotrophic organisms, heterotrophic bacteria distribution, diversity, and metabolic capacity have still been poorly investigated, and isolates with reference genomes from the shallow-sea hydrothermal system are scarce.

In the present work, we revisited the shallow-sea hydrothermal system near Kueishantao Island and collected samples from the water column above the yellow and white vents, in addition to samples of sandy sediments, rocks, and dead vent deposits near the vent sites. Microbial compositions were characterized using culture-independent methods including fluorescence *in situ* hybridization (FISH) and 16S rRNA gene amplicon sequencing. Metagenomic analysis was used to determine the functional and genetic potential of bacteria. The various heterotrophic bacteria with potentially different trophic strategies were isolated from the investigated shallow-sea hydrothermal systems. Some of these isolates may be previously undiscovered bacterial species. Further studies aimed to characterize the physiological and genomic features of bacterial strain P5, a bacterioplankton species with a considerable capacity for adaptation to the shallow-sea hydrothermal environment.

## Materials and Methods

### Sample Collection

**FIGURE 1:**
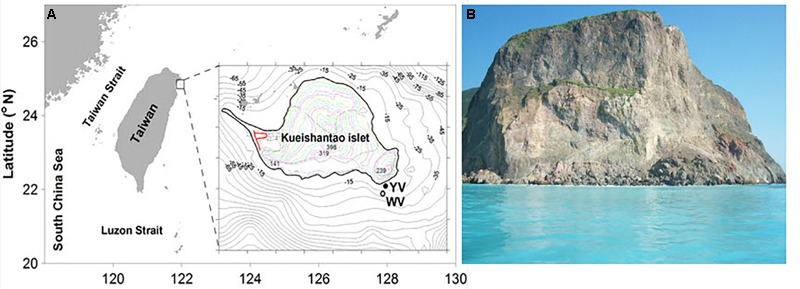
Pictures of the shallow-sea hydrothermal system off Kueishantao Island, Taiwan. **(A)** Map of the sample sites and magnified view of the sample map of the locations indicated by YV (yellow vent area) and WV (white vent area); **(B)** the surface water in the shallow-sea hydrothermal system.

Sampling was performed by scuba divers in May 2015 in two different regions of the shallow-sea hydrothermal system located near Kueishantao Island (N 24.834, E 121.962), Taiwan, China: the white vent area and the yellow vent area (**Figure 1**). All necessary permits were obtained for the described field studies. The vents were observed at depths of approximately 10 and 7 m, respectively. Four samples were collected from the following locations (water samples): W_0m, W_5m, W_surface, and W_outside, which are 0 m above the white vent, 5 m above the white vent, the surface water directly above the vent, and surface water 6 m laterally away from the white vent, respectively. Four more samples were collected from the following locations (water samples): Y_0m, Y_5m, Y_surface, and Y_outside, which are 0 m above the yellow vent, 5 m above the yellow vent, the surface water directly above the yellow vent, and the surface water 6 m laterally away from the yellow vent. The sandy and rocky sediments nearby the yellow vent were named YS_S and YS_R, respectively. The sandy and rocky sediments on the seafloor were named S_S and S_R. The rocky sediment collected nearby the dead vent was named DS_R (an expired vent without the plume and gas discharging). The 2-l water samples were filtered through 3 and 0.2 μm pore-size polycarbonate filters (EMD Millipore Corp., Darmstadt, Germany) for further analysis. Sediment samples were frozen on site and kept frozen during transportation and storage.

### Abundance of Bacteria Determined by CARD-FISH

Fifty-milliliter water samples were immediately fixed with freshly prepared paraformaldehyde (4% final concentration) and stored at 4°C overnight prior to filtration through 0.2 μm pore size polycarbonate filters (Millipore, United States). Filtered samples were stored at -20°C for later analysis by FISH with horseradish peroxidase-labeled oligonucleotide probes (CARD-FISH). Picoplankton abundance was determined by DAPI staining, and bacteria were enumerated by CARD-FISH. Filters were embedded in low-gelling-point agarose and incubated with either lysozyme for the bacteria probe mix (Eub338: 5′-GCT GCC TCC CGT AGG AGT-3′, Eub338II: 5′-GCA GCC ACC CGT AGG TGT-3′ or Eub338III: 5′-GCT GCC ACC CGT AGG TGT-3′) or for the negative control probe (Non338: 5′-ACTCCT ACG GGA GGC AGC-3′) (Teira et al., 2004). Filters were cut into four pieces and hybridized with HRP-labeled oligonucleotide probes and tyramide Alexa488 for signal amplification following the previously described protocol (Teira et al., 2004).

### DNA Extraction

Total DNA of water samples was extracted from the samples with the FastDNA SPIN Kit for Soil (MP Biomedicals, Illkirch, France) according to the manufacturer’s instructions. DNA from the sediment samples was isolated using PowerMax Soil DNA Isolation Kits (MoBio Laboratories Inc., United States) according the manufacturer’s instructions. The concentration and purity of DNA were evaluated using a NanoDrop spectrophotometer (ND-1000, Thermo Fisher Scientific, Waltham, MA, United States). DNA extracts were stored at -80°C until further analysis. The following sequencing was performed at the Chinese National Human Genome Center in Shanghai.

### Amplification and Sequencing of the Bacterial 16S rRNA Gene

Amplicons were generated using fusion degenerate primers 343F (Wilson et al., 1990) and 798R (Rochelle et al., 1995) with ligated overhang Illumina adapter consensus sequences. The libraries were prepared in accordance with the instructions included with the Illumina Nextera XT Index kit (Illumina, United States). Pooled amplicons were purified with the Agencourt AMPure XP purification system (Beckman, United States) and analyzed with an Agilent bioanalyzer 2100 (Agilent Technologies, United States) to confirm appropriate amplicon size. Finally, amplicons were diluted to 10 nM, quantified and sequenced on the Illumina MiSeq platform (reagent kit v.3; Illumina, United States).

### Metagenomes Sequencing

For metagenome sequencing, 1 μg of sample DNA was sheared to 500 bp by Covaris M220 (Covaris, United States). The library was constructed by NEBNext UltraTM DNA Library Prep Kit (NEB, United States). Finally, 10 nM sequencing library was used generate cluster in cBot using TruSeq PE Cluster Kit (Illumina, United States), and sequenced by Illumina Hiseq 2500 for 2 × 250 bp data.

### 16S rRNA Gene Sequence Data Analysis

Acquired Illumina reads were filtered by Meta Genome Rapid Annotation using Subsystem Technology (MG-RAST^1^) QC pipelines to remove the replicated reads (Meyer et al., 2008). The filtered reads were used for the following bioinformatic analysis. For taxonomic analysis, the SILVA small subunit (SSU) database implemented in MG-RAST was used as annotation source for 16S rRNA reads to analyze the bacterial populations in samples using an *E*-value cutoff of 1e-05, minimum identity cutoff of 60%, and minimum alignment length cutoff of 150 bp.

### Shotgun Metagenomic Sequence Data Analysis

Functional profiles were identified using the SEED subsystems annotation source of the MG-RAST, with 1e-05 as maximum *e*-value, a minimum identity of 60%, and a minimum alignment length of 15 amino acids. To remove the bias of average genome size on the sampling of genes from a given metagenomic community, the raw functional gene hits were normalized to the number of *recA* gene encoding recombinase A hits in the respective database. The PRIMER-6 package was used to calculate the Bray–Curtis similarity matrices of metagenomes and generate non-metric multidimensional scaling plots. PERMANOVA implanted in PAST v3.05 was carried out to compare samples from each environment (Hammer et al., 2001). To determine whether the relative abundances of functional genes differed significantly between sample categories, we conducted multiple *t*-tests with *P*-values calculated using a Holm–Sidak correction (Shaffer, 1995) for multiple comparisons implemented in Prism v6.

### Strain Isolation and Culture

All reagents used in bacterial cultures were obtained from Sigma–Aldrich (United States) unless otherwise specified. For the cultivation of bacteria, 200 μL of the water sample was spread onto at least one of the agar plates. The widely used medium for routine culture of marine bacteria were selected (Joint et al., 2010), including low-nutrient R2A (Difco, United States), and nutrient-rich 2216E (Becton-Dickinson, United States), RO (1 g peptone, 1 g yeast extract, 1 g natrium aceticum, 1 g sodium acetate per liter artificial seawater (ASW) with vitamins and trace elements, pH 7.8–8.0), SYPG (30 g NaCl, 0.5 g yeast extract, 0.25 g tryptic peptone, 0.1 g glutamic acid monosodium salt per liter, pH 7.5), SC (1 g D-glucose, 1 g alkapolpeg-600, 1 g L-malic acid, 1 g D-aspartic acid, 1 g yeast extract per liter ASW with vitamins and trace elements, pH7.2–7.5), NS (0.5 g sodium nitrate, 0.5 g sodium sulfate, 0.1 g yeast extract per liter artificial seawater with vitamins and trace elements, pH 7.2–7.5), YTSS (2.0 g yeast extract, 1.25 g tryptone, 20 g sea salt (Sigma) per liter distilled water and autoclave, pH 7.0). All agar plates were incubated at 28°C or under anaerobic conditions at 24°C. Bacterial colonies were picked from the plates and purified further on 2216E plates. The bacterial 16S rRNA gene sequencing and sequence analysis were performed using a previous method (Embley, 1991). The sequence data have been submitted to the NCBI database under accession numbers AM988866 through AM989325. Genomic DNA was extracted from 200 μL of culture using a TIANamp Bacteria DNA Kit (Tiangen, China). The primer pair *pufL*F (5′-CTKTTCGACTTCTGGGTSGG-3′) and *pufM*R (5′-CCATSGTCCAGCGCCAGAA-3′) were designed to amplify *pufL* and *pufM* through PCR to allow detection of the photosynthetic reaction center genes in the strains (Cho et al., 2007).

### Genome Sequencing and Analysis

Whole genome sequencing of strain P5 was accomplished using a hybrid approach, combining Illumina short read data with PacBio long read data (Koren et al., 2012). For PacBio sequencing, 5 μg of sample DNA was sheared to 10 Kb by a Covaris^®^ g-TUBE^®^ (Covaris, United States). A PacBio^®^ SMRTbell^TM^ Template Prep Kit (PacBio, United States) was used to construct the library. The sequencing primers were annealed using a PacBio DNA/Polymerase Kit (PacBio, United States) and polymerase combined with the SMRTbell templates. We obtained long read data from PacBio RS II PacBio RS II (PacBio, United States). The raw Illumina data were filtered by the FASTX-Toolkit to remove the adapters, N bases, and low-quality reads. The clean data were assembled using Velvet v1.2.03 with default parameters. The PacBio long reads were assembled by RS HGAP assembly 3. The complete genome was finally gap closed by Sanger sequencing. The final assembled genomes of P5 were automatically annotated and analyzed through the IMG/ER^2^. The comparison and visualization of multiple genomes was conducted with BRIG (Alikhan et al., 2011).

### Physiological and Biochemical Analysis of Strain P5

Strain P5 was isolated from surface seawater off Kueishantao Island, northeast Taiwan by 2216E medium. The strain was adaptively grown for 3 days (inoculated into a new bottle everyday) in ASW containing a 1 mM concentration of dissolved organic carbon (DOC). The DOC concentration was adjusted by ASW supplemented with full strength medium (5 g peptone and 1 g yeast extract per liter ASW and the final concentration was determined using a total organic carbon analyzer (Shimadzu, Japan). Bacteria (10^5^ cells/mL) were then washed three times with autoclaved ASW and used to inoculate growth medium consisting of artificial seawater (ASW) base combined with substrates including 0.45 μM DOC, 2.5 mM NaHCO_3_, and 1 mM Na_2_S_2_O_3_ in phosphate-buffered saline (pH 7.4) (autotrophic culture: NaHCO_3_ and Na_2_S_2_O_3_; heterotrophic culture: DOC). Cultures were incubated at 22°C at 160 rpm/min in the darkness or light (12 h)/dark (12 h) cycle for 4 days. To determine bacterial cell density, cultures were stained with SYBR Green I (1:100 dilution; Molecular Probes, United States) for 15 min, and measured via flow cytometry (BD Accuri C6, United States). All culture experiments were performed in triplicate.

The strain P5 was cultured in 2216E and freeze-dried by a Freeze Dry System (Labconco Corp., Czechia) for 48 h, a moderate amount of chloroform was added, and cells were broken under a Ultrasonicator SM-650D (Shunma, China). The Ultrasonicator was operated for a total of 10 min containing 9 s for running, and 5 s for stopping. The power was 22% (total power 650 W). The supernatant was measured using a UV-Vis spectrophotometer (Agilent 8453, United States) after being centrifuged at 7006 g/min for 10 min. Bacteriochlorophyll *a* was measured with a FIRe Fluorometer Induction and Relaxation System (Satlantic, Canada) at 800 nm.

### Nucleotide Sequence Accession Numbers

The metagenomic datasets are publicly available in the MG-RAST system under project identifiers W_0m (4671689.3 and 4668298.3), W_5m (4671690.3 and 4668304.3), W_surface (4671687.3 and 4668303.3), W_outside (4671688.3 and 4668305.3), Y_0m (4671691.3 and 4668302.3 for 16S rDNA and functional metagenome, respectively), Y_5m (4671692.3 and 4668301.3), Y_surface (4671693.3 and 4668300.3), Y_outside (4671694.3 and 4668299.3), YS_S (4647123.3 and 4644370.3), YS_R (4647124.3 and 4644369.3), S_S (4647121.3 and 4644371.3), S_R (4647122.3 and 4644368.3), and DS_R (4647120.3 and 4644372.3). They have also been deposited in the NCBI Short Read Archive: 16S rDNA reads, SRR5229862-SRR5229869 for W_0m, W_5m, W_surface, W_outside, Y_0m, Y_5m, Y_surface, and Y_outside, respectively, YS_S (SRR5149598), YS_R (SRR5149595), S_S (SRR5149599), S_R (SRR5149606), DS_R (SRR5149594), metagenomic DNA reads, SRR5229878-SRR5229885 for W_0m, W_5m, W_surface, W_outside, Y_0m, Y_5m, Y_surface, and Y_outside, respectively, YS_S (SRR5149596), YS_R (SRR5149597), S_S (SRR5149607), S_R (SRR5149602), DS_R (SRR5149604). The complete genome sequence of strain P5 has been deposited into GenBank under the accession numbers CP015039 (chromosome) and CP015040-CP015043 (plasmids).

## Results and Discussion

### Environmental Parameters

The fluids in the shallow-sea hydrothermal system were slightly acidic (approximately pH 6) and oxic (Supplementary Table S1). Salinity, dissolved inorganic carbon, nitrate, nitrite, and phosphate concentrations were nearly oceanic (Supplementary Table S1). Elemental sulfur (S^0^) is naturally enriched in the shallow-sea hydrothermal fluids near Kueishantao Island. Compared to deep-sea vents, the sulfide concentration was lower in this shallow vent system (Chen et al., 2005; Chan et al., 2016). Compared to the common marine environment, the concentartion of DIC was higher, while the DOC centration was similar in the shallow-sea hydrothermal system (Supplementary Table S1). These physico-chemical properties of the hydrothermal system might support not only autotrophic bacteria, but also heterotrophic bacteria.

### Cell Densities

The DAPI-based total cell counts were on average on the order of 10^6^ cells mL^-1^ (Supplementary Table S2). The general bacterial probe mixture EUB338 hybridized 90–94% of all DAPI-stained cells in the white vent area, while the percentage of bacterial probe in the yellow vent area was 57–87% (Supplementary Table S2). Compared to the white vent (temperature up to 58°C inside the vent), the relatively high temperature (up to 116°C) near the yellow vent might result in lower bacterial abundance and their proportions of total microbial cells at the Y_0m site (0 m above the yellow vent) (only 57%).

### Bacterial Community Composition

**FIGURE 2:**
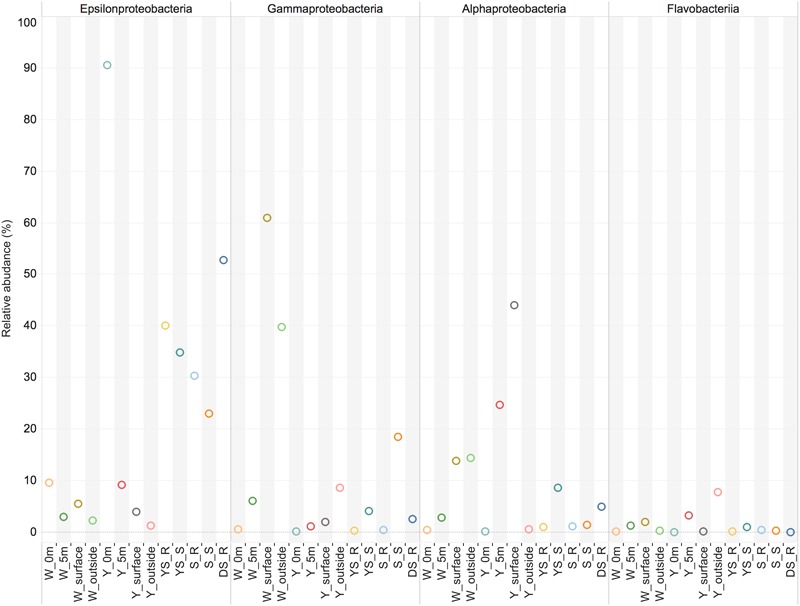
The main class-level bacterial composition among the samples. The map plot depicts the relative percentage of each bacteria within each sample. Water samples: W_0m, W_5m, W_surface, W_outside, Y_0m, Y_5m, Y_surface, and Y_outside; sediment samples: YS_S, S_R, S_S, S_R, and DS_R.

The investigation of the microbial communities using 16S rRNA gene amplicon sequencing revealed that a significant proportion of the sequences in the water samples (14–89%) and sediment samples (21–47%) could either not be unassigned or were assigned to unclassified groups, indicating the presence of so far uncharacterized bacteria within this geothermal ecosystem. Campylobacteria were apparently present in relatively high abundance in the sediment samples, and represented the main bacterial groups at the Y_0m site and the W_0m 318 site (water samples, 0 m above the vents) (**Figure 2**). However, the relative abundance of 16S rRNA sequences of Campylobacteria decreased in the water column above the vents (**Figure 2**). Few sequences of Campylobacteria were classified into those from sulfur-reducing chemolithoautotrophs *Nautiliales*-like organisms that were previously known to be a dominant group in the white vent (Zhang et al., 2012; Tang et al., 2013). The shallow-sea hydrothermal vents varied greatly in chemical composition of gasses discharging such as CO_2_ and H_2_S (Chen et al., 2016). In the previous one, the DIC concentrations was about 6 mM in 0 m above the white vent (Zhang et al., 2012), however, there was only a little higher (∼2.7 mM) than that at nearby seawater in this study. On the other hand, the H_2_S concentrations have declined since 2014 (Chen et al., 2016). In addition, physical conditions such as tide could influence the hydrothermal ecosystem (Chen et al., 2005). These might influence on the microbial community composition. Gammaproteobacteria were found abundantly at the surface waters and outside the white vent (W_surface site and W_outside site) (**Figure 2**). However, the very few sequences of sulfur-oxidizing Gammaproteobacteria *Thiomicrospira*-like organisms were detected. Members of the family *Alteromonadaceae* and *Oceanospirillaceae* within Gammaproteobacteria are the most abundant organisms in W_surface and W_outside, respectively. Y_5m (5 m above the yellow vent) and Y_surface (the surface water immediately above the yellow vent) have abundant members of Alphaproteobacteria (**Figure 2**). Flavobacteriia were present at all sites (**Figure 2**). There are some relatively rare taxa found in the hydrothermal system including Deltaproteobacteria, Beatproteobacteria, Actinobacteria, Cyanobacteria, Aquificae, Chorobi, Spirochaetes, and Tenericutes. Deltaproteobacteria, and Firmicutes are mainly found in sediments samples (**Figure 2**).

Overall, homology-based taxonomic assignments of shotgun metagenomics data predicted by the MG-RAST showed that Gammaproteobacteria and Alphaproteobacteria were abundant classes in the white and yellow vent water samples, making up approximately 60% of the microbial assemblage, whereas Campylobacteria were abundant class in the vent sites and sediment samples. The most dominant proteobacterial orders overall were *Rhodobacterales* and *Rhizobiales* within the class Alphaproteobacteria; *Alteromonadales, Pseudomonadales*, and *Oceanospirillales* within the class Gammaproteobacteria. However, many shotgun metagenomic sequences (mostly > 50% of the total sequences) still remained functionally unannotated and taxonomically unassigned due to the limitations of the available tools and the paucity of reference genomes. These could possibly lead to a discrepancy between shotgun metagenomics data taxonomic assignment and 16S rRNA-based classification.

### Metabolic Potentials and Functional Biomarkers of Microbial Communities

**FIGURE 3:**
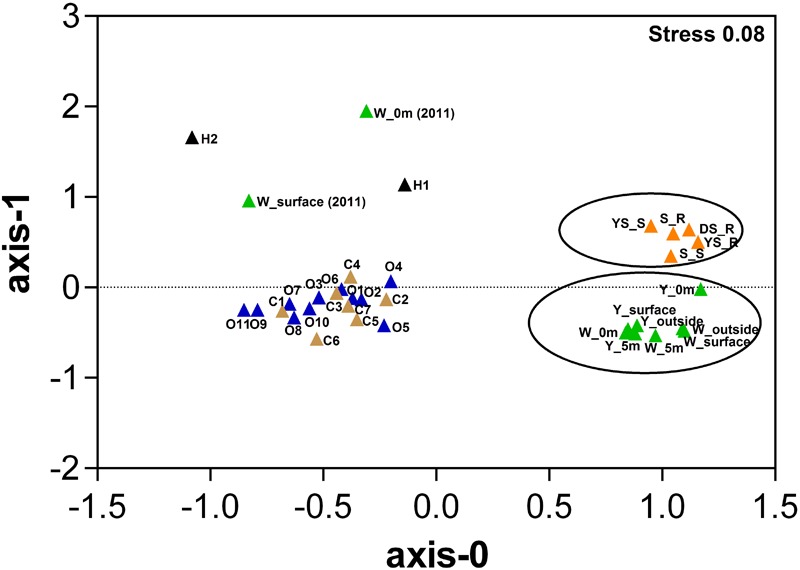
Multidimensional scaling plots of samples using Bray–Curtis similarity according to the SEED subsystem. Color represents different sample areas and each habitat label type (n) is indicated [data highlighted by two circles from this study; W_0m (2010) and W_surface (2010) data from our previous study (4) (Tang et al., 2013), H1 and H2 data from references (Grzymski et al., 2008; McDermott et al., 2015), other data from a reference (Rusch et al., 2007), Details in Supplementary Table S3. Samples from each of the respective environments clustered together based on their functional profile. The stress values are reported in the top right corner and represent the goodness-of-fit.

Based on the relative abundance of SEED subsystems, multidimensional scaling plots show that the hydrothermal water samples in this study clustered together at the functional level, and were separate from the hydrothermal sediments samples (**Figure 3** and Supplementary Table S3); permutational multivariate analysis of variance (PERMANOVA) indicates that this difference is significant (*P* = 0.009). We observed a set of SEED functions “Carbohydrates” and “Amino Acids and Derivatives” that contributed strongly to the difference between the communities in the sediments and water samples; that set of SEED functions is more abundant in the hydrothermal water samples. Compared to a metagenome from the previous investigation in the white vent area, “Carbohydrates” were more abundant in the metagenomic datasets in this study, but there were fewer “Protein metabolism” results. Compared to the bacterial community compositions from the previous investigation, Flavobacteriia were more abundant in this study. The Flavobacteriia genomes contain diverse and abundant glycoside hydrolases (GHs) that are involved in carbohydrate metabolisms (Tang et al., 2017). Two categories, “Carbohydrates” and “Protein metabolism,” together contributed to approximately 26% of the differences between communities in the two metagenomes. However, “Sulfur metabolism” and “Photosynthesis” contribute less to their differences. The hydrothermal water samples were functionally distinct from those in the costal and open seawater samples (PERMANOVA, *P* < 0.01 in all cases). The genes associated with the cell wall and capsule category, and virulence, disease, and defense category were more abundant in the hydrothermal water samples than those in the costal and open seawater samples metagenomes, which are main contributors to the dissimilarity between metagenomes (Supplementary Table S3).

**FIGURE 4:**
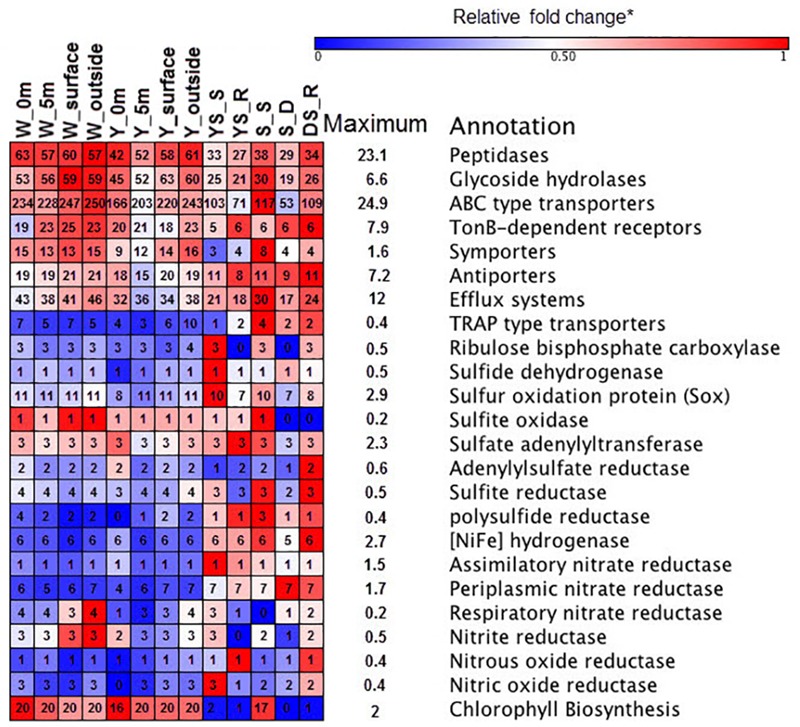
Heat map of functional composition among the samples. The value of functional gene relative to *recA* (single copy control gene) is assigned with a color relative to the maximum value among all comparisons of each gene. The colors represent the minimum (blue), middle (white, 0.5), and maximum (red) values listed on the right. The number in the box represented the components of each functional category.

The functional metagenomes suggested that oxidation of reduced sulfur compounds in the hydrothermal system could occur through the Sox multienzyme system (**Figure 4**), which catalyzes the complete oxidation of reduced sulfur compounds to sulfate (Yamamoto and Takai, 2011). Several enzymes have been proposed to as possible oxidizers of inorganic sulfur compounds, including sulfide quinone oxidoreductase and sulfite oxidase, which oxidize sulfide to elemental sulfur and sulfite to sulfate, respectively (**Figure 4**) (Yamamoto and Takai, 2011). Genes encoding polysulfide reductase (*Psr*) are present in the sediments metagenome, resulting in the reduction of polysulfide derived from elemental sulfur to sulfide (Yamamoto and Takai, 2011). Previous metagenome analyses have suggested that Campylobacteria in the vent can gain energy from sulfur-reduction catalyzed by Psr to fix CO_2_ by the rTCA cycle (Tang et al., 2013). However, the relative gene abundance of *Psr* in the water samples was lower than those in the sediments (**Figure 4**). The Deltaproteobacteria contributed to *Psr* gene sequences in the sediment metagenomes. In addition, few key genes encoding ATP-dependent citrate lyase for the rTCA cycle (Hügler and Sievert, 2011) were found in any of the metagenome datasets in this study, whereas genes encoding ribulose-1,5-bisphosphate carboxylase (RuBisCO) were found; those genes mediated the CBB cycle for carbon fixation. The relative abundances of *RuBisCO* (normalized to *recA* genes) in the hydrothermal water samples, on average, were 0.12 and 0.14 in the yellow vent and white vent areas, respectively, which were both lower than found (0.98) in a previous investigation (Tang et al., 2013). In contrast, heterotrophic metabolism might be predominant both in the water column and the sediment, as indicated by a set of transporters, peptidases, and GHs genes in all the metagenomic datasets (**Figure 4**). Although the relative abundances of genes encoding GH and peptidase between the sediment samples and water samples display no statistical difference (*t*-test, *P* > 0.01), they are more diverse in the waters than in the sediment of hydrothermal system, indicating a wider spectrum of substrate utilization for bacteria in the waters of this shallow-sea system. Similarly, more ATP-binding cassette systems (ABC transporters), symporters, and TonB-dependent receptors (TBDTs) were found in the hydrothermal waters, which allowed them to import organic matter efficiently (**Figure 4**). All metagenomes were found to possess exporters involved in the efflux of heavy metals and metabolites (Supplementary Table S4). Predicted substrates for the transport systems in the metagenomes include a variety of carbohydrates, carboxylic acids, amino acids, peptides, metals, and other nutrients (Supplementary Table S4).

All metagenomes were found to contain genes encoding sulfate adenylyltransferase, adenylsulfate kinase, and adenylsulfate reductase, which are required for assimilatory sulfate reduction to supply sulfur for biosynthesis in aerobic marine bacteria (**Figure 4**). Functional metagenomic analyses indicated that the relative abundance of genes encoding Psr, Ni-Fe hydrogenase, and periplasmic nitrate reductase exhibited statistically significant differences between hydrothermal sediments and water samples (*t*-test, *P* < 0.05) and it was more abundant in sediment samples. The genes encoding Ni–Fe hydrogenase in the metagenome enabled bacteria to use H_2_ as an energy source (Petersen et al., 2011). There is some possibility that nitrate could be used as alternative electron acceptor in the presence of periplasmic nitrate reductase (Canfield et al., 2010). Thus, chemolithotrophs might contribute to the chemical transformations of elements in the sediment.

Overall, the *Rubisco* gene sequences were mainly homologous to those in the order *Rhodobacterales, Rhizobiales, Methylococcales*, and *Thiotrichales*, in contrast to previous metagenomes where most of the *Rubisco* gene sequences homologs were affiliated with *Thiomicrospira*-like (Tang et al., 2013). Cyanobacteria also contribute to *Rubisco* gene pool at the sea surface of the hydrothermal system. The order *Desulfovibrionales* within Deltaproteobacteria in the vent sites and sediment samples contributed to genes encoding NiFe hydrogenase. The majority of genes encoding TBDTs were closely related to Flavobacteriia and Gammaproteobacteria (primarily members of the *Alteromonadales* and the *Pseudomonadales*) in the water samples, while TBDTs sequences from the Campylobacteria and Deltaproteobacteria class were found in sediment samples. The members of the order *Rhodobacterale* contributed greatly to the diversity of ABC transporters in the metagenomes, and the members of Flavobacteriia contribute greatly to the diversity of peptidases and glycoside hydrolases in the metagenomes. The sox sequences in the metagenomic data were mainly homologous to those found in the orders *Rhodobacterale* and the class Campylobacteria. Genes encoding periplasmic nitrate reductase and dissimilatory nitrite reductase were more prevalent in the sediment dataset than in the water sample dataset, and they were mainly homologous to sequences from the members of Campylobacteria and Deltaproteobacteria.

### Taxonomic Assignment of the Isolated Strains

**FIGURE 5:**
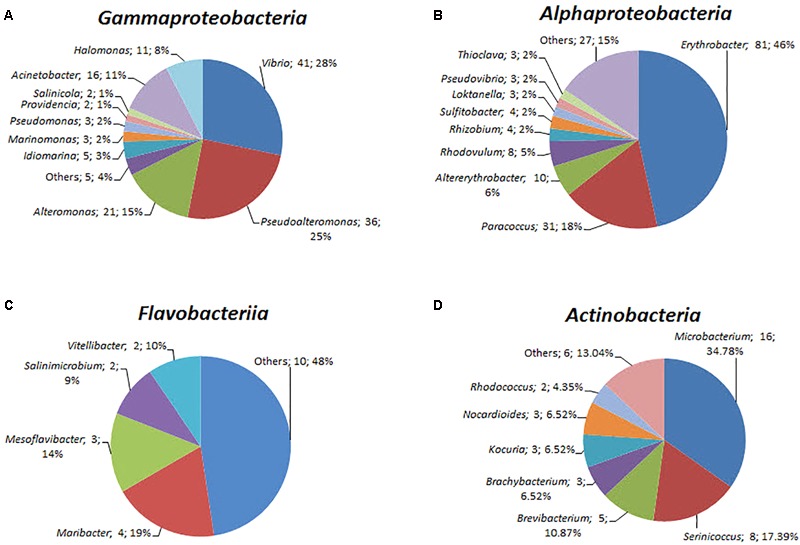
The diversity of the bacterial isolates. **(A)** Gammaproteobacteria, **(B)** Alphaproteobacteria, **(C)** Flavobacteriia, and **(D)** Actinobacteria. Each color represents the percentage of the taxon in the total isolates.

A total of 408 isolates were identified by near full-length 16S rRNA gene sequence analysis (Supplementary Table S5). Of these isolates, 187 were from the yellow-vent area, 111 were from the white-vent area, and 65 were from the surface water above the vents. It was noted that 42 isolates were obtained from the waters above the dead vent. A previous study showed that heterotrophic bacteria dominated inactive deep-sea hydrothermal system (Sylvan et al., 2012). The isolates were distributed into seven different bacterial classes from four different phyla, namely Proteobacteria, Bacteroidetes, Actinobacteria, and Firmicutes. However, the failure to isolate Epsilonprobacteria strains may be because the optimal conditions for their culture have yet to be established. The majority of the characterized strains belonged to the Alphaproteobacteria (174 strains), Gammaproteobacteria (145 strains), and Actinobacteria (46 strains). The rest of the strains were assigned to Betaproteobacteria, Flavobacteriia, *Cytophaga*, and *Bacillus*. The spectrum of different genera was greatest within the classes Alphaproteobacteria (31 genera), followed by Gammaproteobacteria (15 genera), Flavobacteriia (14 genera), and Actinobacteria (12 genera) (**Figure 5**). For the classes Alphaproteobacteria, *Erythrobacter* (81 strains) and *Paracoccus* (31 strains) were the predominant genus (**Figure 5**). For the classes Gammaproteobacteria, *Vibrio* (41 strains) and *Pseudoalteromonas* (36 strains) constituted a high proportion (**Figure 5**). For the classes Actinobacteria, *Microbacterium* (16 strains) was dominant. These taxonomic groups are commonly detected in ocean environments (Haggerty and Dinsdale, 2017).

These isolates may possess novel functions and exhibit a broad range of ecological attributes and life-history strategies. For example, some strains belonged to oligotrophic taxa (including members of *Erythrobacteraceae* and *Sphingomonadaceae* groups); some strains belonged to the copiotrophic taxa (including members of *Vibrio* and *Alteromonadaceae* phyla) and may be able to rapidly consume labile DOC (Church, 2009; Kirchman, 2015). The members of *Flavobacteriaceae* have the ability to degrade complex high-weight molecule organic compounds (Buchan et al., 2014). Those in the genera *Erythrobacter* and *Novosphingobium* can to metabolize nutrient-poor and recalcitrant carbon substrates (Church, 2009; Kirchman, 2015). Furthermore, *pufL* and *pufM* genes, which encode photoreaction center L and M polypeptides, respectively, in *Erythrobacter* were identified as aerobic anoxygenic phototrophic bacterial gene biomarkers. Previous studies have suggested that they have the capacity to undergo photoheterotrophy in marine environments (Jiao et al., 2007).

**FIGURE 6:**
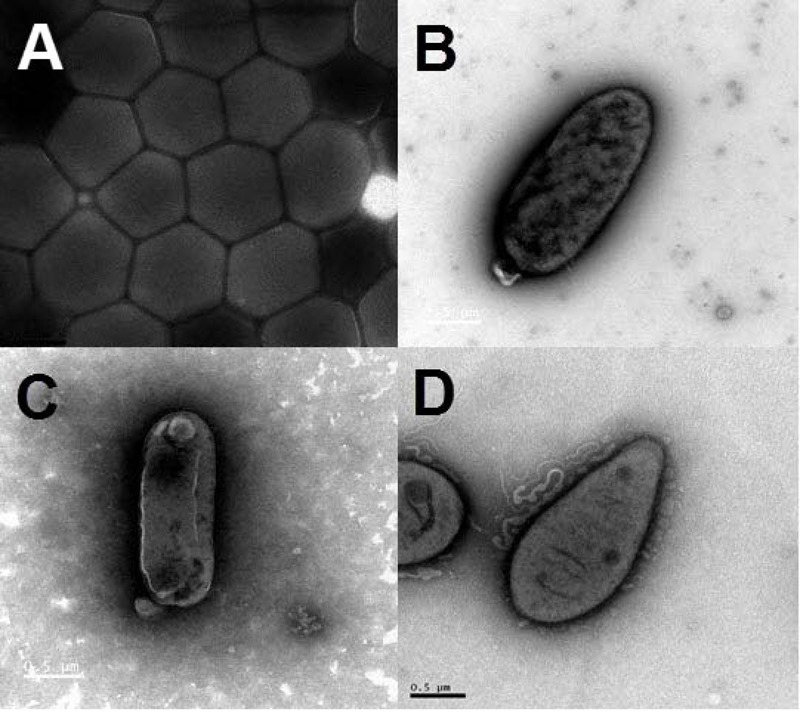
Transmission electron micrograph images of representative strains isolated from the shallow-sea hydrothermal system. **(A)** Strain JLT9 belonging to the genus of *Serinicoccus* within Actinobacteria, **(B)** Strain T28 belonging to the genus of *Maribacter* within Flavobacteriia, **(C)** Strain JL3646 belonging to the genus of *Marivivens* within Alphaproteobacteria, **(D)** Strain P5 belonging to the genus of *Rhodovulum* within Alphaproteobacteria.

On the basis of the taxonomic assignment by the EzTaxon classifier using annotated 16S rRNA gene sequences (Chun et al., 2007), a total of 58 strains from the classes Alphaproteobacteria, Gammaproteobacteria, Flavobacteriia and Actinobacteria might represent new bacterial species not yet validly described with less than 98% maximum identity with their closest BLAST hits (Supplementary Table S5). Among them, the information on the first complete genome of a cultivated actinomycete strain JLT9 isolated from the shallow-sea hydrothermal system was reported, which contained various sulfur oxidation genes (**Figure 6A**) (Han et al., 2017). The complete genome of the *Maribacter* sp. T28 harbored the xylanolytic, alginolytic and pectinolytic enzymes responsible for polysaccharide degradation (**Figure 6B**) (Genbank accession, CP018760) (Zhan et al., 2017). The genome data suggested that *Marivivens* sp. JLT3646 within Alphaproteobacteria has the potential to degrade aromatic monomers (**Figure 6C**) (Genbank accession, CP018572 and CP018573 for chromosome and plasmid, respectively) (Chen et al., 2017).

**FIGURE 7:**
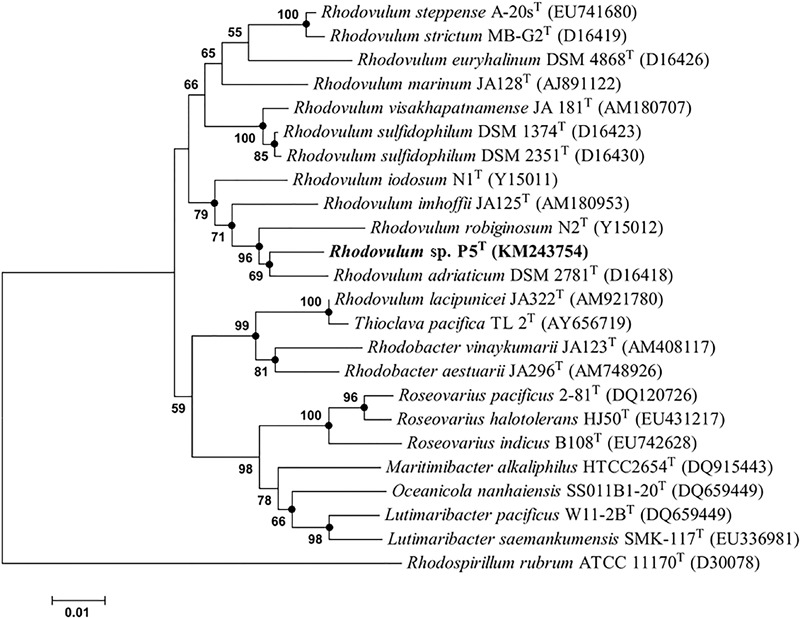
Phylogenetic tree based on alignment of 16S rRNA gene sequences showing the relationship of strain P5 (shown in bold print) within the genus *Rhodovulum*. The trees were constructed by the Neighbor-Joining method using MEGA version 6.0 software (closed circles represent the same tree branch using the algorithm of Maximum Likelihood and Maximum Parsimony) and rooted by using *Rhodospirillum rubrum* ATCC 11170^T^ as the outgroup. Numbers at nodes represent bootstrap values (based on 100 resamplings). Bootstrap values more than 50% are noted. The GenBank accession numbers for 16S rRNA gene sequences are shown in parentheses. Bar, 1 nt substitution per 100 nt.

The gene sequences homologs affiliated with *Rhodobacterales* were abundant, accounting up to 20.57% of the total sequences in the water sample. Strain P5 (**Figure 6D**) exhibited a 97.48% 16S rRNA sequence similarity with *Rhodovulum adriaticum* DSM 2781 within the order *Rhodobacterales* (**Figure 7**). Total of eight strains of *Rhodovulum* were isolated form the white and yellow vents area (Supplementary Table S5). The members of *Rhodovulum* were also frequently found near the shallow-sea submarine vents of Panarea Island (Maugeri et al., 2013a). The newly discovered strain P5 of great metabolic versatility could be considered to be representative of heterotrophic bacteria isolate from the shallow-sea hydrothermal system.

### Biochemical and Physiological Characteristics of Strain P5

**FIGURE 8:**
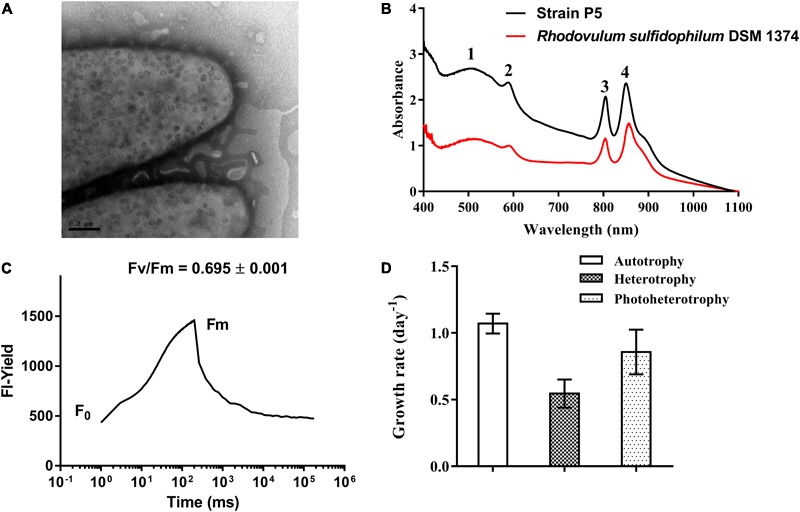
Morphological, biochemical and physiological characterization of bacterial strain P5. **(A)** Transmission electron micrograph images of strain P5. The external surface of the cytoplasmic membrane of strain P5 is of vesicle type. **(B)** *In vivo* absorption spectrum of strains P5 and reference strain. **(C)** Fluorescence induction and relaxation kinetics recorded by infrared fluorometry. **(D)** Growth rates of bacteria.

Strain P5 is a Gram-negative, spindle-shaped, purple bacterium (1.5–2.0 μm in length and 0.9–1.0 μm in width) (**Figure 6D**) that can grow at the temperature range of 20–40°C (optimum, 26–34°C), in the pH range of 5–9 (optimum, 6–8), in the presence of 0–6.0% (w/v) NaCl (optimum, 3.0%), and in aerobic or microanaerobic conditions (Supplementary Table S6). Electron microscopy of ultrathin sections revealed the presence of a vesicular type internal photosynthetic membrane that is a common feature in other *Rhodovulum* species (**Figure 8A**) (Masuda et al., 1999; Srinivas et al., 2007; Kompantseva et al., 2010; Maugeri et al., 2013a). The *in vivo* absorption spectrum of intact P5 cells exhibited four major peaks at 508, 588, and 804 and 850 nm, thus confirming the presence of bacteriochlorophyll *a* and carotenoids (**Figure 8B**). The functionality of the photosynthetic apparatus was investigated by infrared kinetic fluorescence measurements. P5 displayed clear induction of bacteriochlorophyll *a* with F_V_/F_M_ 0.749 ± 0.003 (mean ± SD; *n* = 3), which confirmed that its fully functional photosynthetic reaction centers are connected to an efficient electron transfer chain (**Figure 8C**). These results suggested that P5 had phototrophy capability similar to other *Rhodovulum* species (Masuda et al., 1999; Srinivas et al., 2007; Kompantseva et al., 2010; Maugeri et al., 2013a). Strain P5 is capable of heterotrophic, autotrophic and photoheterotrophic growth (**Figure 8D**).

### Genomic Features of Strain P5

**FIGURE 9:**
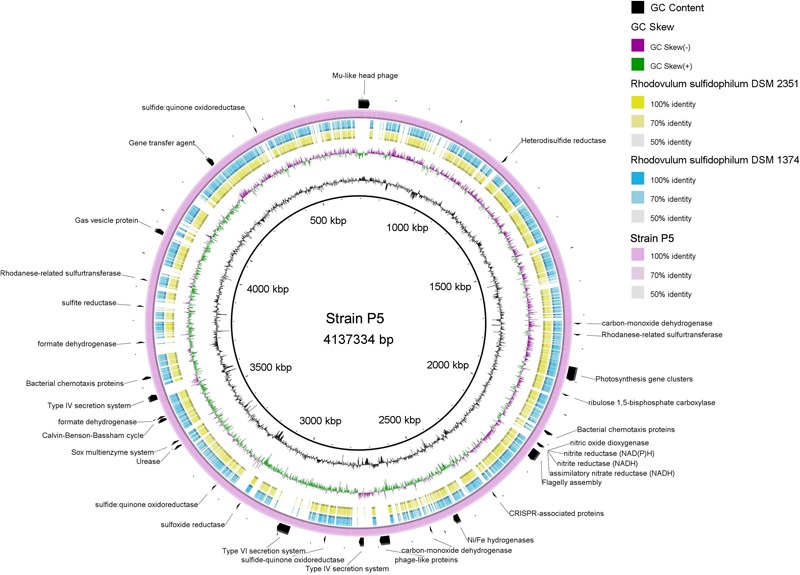
Multiple genome comparison of strain P5 (chromosome) and *Rhodovulum sulfidophilum* strains. The composite genome comparison Figure was generated using BRIG after performing a BLASTn analysis of strain P5 as the reference genome. Each genome mapping to the reference is represented as a colored ring, with a solid color representing greater than 50% sequence identity. The innermost rings show GC content (black) and GC skew (purple/green) in strain P5. The remaining rings indicate genome regions of *R. sulfidophilum* DSM 2351 (green), *R. sulfidophilum* DSM 1374 (blue), and strain P5 (purple). The predicted major functional genes in P5 are labeled on the outermost ring with black arcs.

Strain P5 exhibited a genome of 4,137,334 bp (one chromosome and four plasmids) with a G+C content of 64.64% that coded 4,243 protein-coding and 68 RNA genes (including nine rRNA operons). Bidirectional BLAST analyses showed that approximately 3,000 genes exhibited > 50% sequence identity between strain P5 and two sequenced genomes of *Rhodovulum* species [*Rhodovulum sulfidophilum* DSM 1374 (Nagao et al., 2015b) and *Rhodovulum sulfidophilum* DSM 2351 (Nagao et al., 2015a)] (**Figure 9**), indicating identical or equivalent function.

Strain P5 was predicted to possess complete central carbon metabolic pathways, including glycolysis, the pentose phosphate pathway, the Entner–Doudoroff pathway, and the tricarboxylic acid cycle. The P5 genome harbored genes encoding 16 GHs and 30 peptidases as well as a urease gene cluster, indicating the potential for the degradation of carbohydrates, proteins, peptides, and urea (Supplementary Table S7). The most abundant transporter systems in the P5 genome were ABC transporter components, followed by TRAP-type transport components, and the exporters and antiporters, accounting for 8.1% of the total protein-coding genes of the chromosome in P5. Predicted uptaking substrates for the complete transporter systems comprised a variety of carbohydrates, carboxylic acids, amino acids, peptides, alkanesulfonate, metals, and other nutrients. The genome harbored two genes sets encoding type IV secretion machinery systems that are used to deliver proteins and DNA into the extracellular environment, whereas the other two *Rhodovulum* species lack these genes (**Figure 9**). This organism has genes for the complete CBB required for autotrophic carbon fixation. Well-known electron donors utilized for microbial autotrophic growth are hydrogen and reduced forms of sulfur (sulfide, S^0^, and thiosulfate). Strain P5 possessed the complete repertoire of genes for the oxidation of reduced sulfur compounds, which increased niche-specialization competitiveness in the shallow-sea environments. These genes encoded enzymes for the oxidation of reduced sulfur compounds including the Sox enzyme complex for oxidation of reduced sulfur to sulfate (SO_4_^2-^), sulfide quinone oxidoreductase, mediating the oxidation of sulfide to elemental sulfur, rhodanese sulfurtranferase for oxidation of thiosulfate (S_2_O_3_^2-^) to sulfite (SO_3_^2-^), and reverse dissimilatory sulfite reductase for oxidation of elemental sulfur to sulfite, adenosine 5-phosphosulfate reductase and sulfate adenylyltransferase for oxidation of sulfite to sulfate (SO_4_^2-^). Strain P5 has two gene clusters encoding different Ni–Fe hydrogenases, which is predicted to catalyze the reversible oxidation of hydrogen gas and enables bacteria to use molecular hydrogen as a source of energy. The heterodisulfide reductase-related proteins (Hdr) are only identified in the P5 genome (**Figure 9**), and are likely candidates to be involved in energy coupling through electron bifurcation from diverse electron donors such as formate or H_2_ via formate dehydrogenase or Hdr-associated hydrogenase (Kaster et al., 2011). Similar to other *Rhodovulum* species, the genome of strain P5 also contains all of the genes required for photosynthesis gene clusters (PGCs), including those involved in biosynthesis of bacteriochlorophyll *a*, carotenoids, light-harvesting systems, and reaction center components (Supplementary Table S7).

The genomes of P5 contain one large gene set that encodes a flagellum system and genes that encode methyl-accepting chemotaxis proteins, indicating that P5 may use them to facilitate their movement toward nutrient rich zones. Genes encoding gas vesicle proteins have been identified, providing buoyancy to cells as flotation devices in order to obtain the optimum amount of light and nutrients at a suitable depth in the environment. Several chemotaxis proteins were located near the gas vesicle proteins (*gvp*) gene cluster. In contrast, two *Rhodovulum* species lack the *gvp* gene cluster (**Figure 9**).

Strain P5 carries several prophage-like elements, in which a Mu-like head group phage has been induced successfully (Lin et al., 2016). The presence of clustered regularly interspaced palindromic repeat (CRISPR) arrays and their associated *Cas* genes in P5 and two other *Rhodovulum* species form a system that is possibly involved in bacterial defense against phages or plasmids. However, CRISPR sequences revealed no similarity between strains with regards to the numbers of repeats and spacer sequences, indicating that the histories of phage infection are different. Both bacteria have a predicted gene transfer agent (Supplementary Table S7).

With respect to morphological and biochemical traits as well as phylogenetic relationships and genomic analysis, isolate P5 is clearly a strain belonging to the genus *Rhodovulum*.

### Summary

In this study, few 16S rRNA gene sequences were related to *Thiomicrospira-* and *Nautiliales*-like chemolithoautotrophic bacteria, both of which were previously reported to be abundant in the investigated Kueishantao shallow-sea hydrothermal system (Zhang et al., 2012; Tang et al., 2013). In contrast, heterotrophic bacteria in the investigated hydrothermal system were abundant. This hints at substantial spatial and/or temporal variability in the composition of the microbial communities of the shallow-sea hydrothermal system could be enormous. The genetic potential of the microbial community was analyzed using marker genes for the carbon, nitrogen, and sulfur metabolism. In the present investigation, we detected the potential for chemotrophic CO_2_ fixation mainly through the CBB cycle. Sulfur oxidation and reduction marker genes were present. Genes encoding enzymes involved in the denitrification pathway and H_2_ utilization were also detected. The metagenomes contained abundant genes responsible for heterotrophic utilization of organic substrates including transporters, glycoside hydrolases, and peptidases genes. Cultivation attempts targeting heterotrophs resulted in the isolation of 408 heterotrophic strains that are typically considered as oligotrophic, copiotrophic, or phototrophic bacteria, in which a novel species *Rhodovulum* sp. P5 was isolated. Physiological analysis indicated that P5 is capable of heterotrophy, autotrophy, and phototrophy. The P5 genome possessed the complete CBB cycle, PGCs, Ni-Fe hydrogenases, and the complete repertoire of genes involved in the oxidation of reduced sulfur compounds that are putative metabolic potentials of heterotrophs associated with the adaptions to the shallow-sea hydrothermal system. The genome harbored two gene sets encoding type IV secretion machinery systems, the *gvp* gene cluster, and the genes encoding heterodisulfide reductase that were not present in two *Rhodovulum* isolates from other habitats, which increased niche-specialization competitiveness in the shallow-sea environments. This study unveiled that the shallow-sea hydrothermal system harbored diverse microbial communities and their potential functions, enabling us to conduct more focused studies on heterotrophic activity *in situ* and ecophysiological features of isolated bacteria to ultimately get a complete picture on the tight coupling between microbes and biogeochemical cycling in the shallow-sea hydrothermal ecosystems in the future.

## Author Contributions

KT and NJ conceived and designed the experiments. YZ, DL, YH, C-TC, DW, Y-SL, JS, and QZ conducted the experiments. KT and YZ analyzed the data. All of the authors assisted in writing the manuscript, discussed the results, and commented on the manuscript.

## Conflict of Interest Statement

The authors declare that the research was conducted in the absence of any commercial or financial relationships that could be construed as a potential conflict of interest.

## Footnotes

1 http://metagenomics.anl.gov/

2 http://img.jgi.doe.gov
