# Supplementary material for: Cultivation-Independent and Cultivation-Dependent Analysis of Microbes in the Shallow-Sea Hydrothermal System Off Kueishantao Island, Taiwan: Unmasking Heterotrophic Bacterial Diversity and Functional Capacity
Source: Front Microbiol. 2018 Feb 22;9:279. doi: 10.3389/fmicb.2018.00279 (PMC5829616; doi:10.3389/fmicb.2018.00279)
Supplement: Supplementary file 2 [file Table_2.DOC]

**Table S2.** DAPI-stained and EUB338 hybridized cells in the shallow-sea hydrothermal system in Kueishantao Island (mean ± S.D., N=4).

| **Station** | **DAPI**  **(****10^6^ cells ml^-1^)** | **EUB338**  **(10^6^ cells ml^-1^)** | **EUB338 / DAPI**  **(%)** |
| --- | --- | --- | --- |
| W_0m | 4.51±0.06 | 4.07±0.05 | 90.24±2.31 |
| W_5m | 5.15±0.13 | 4.84±0.08 | 93.90±3.92 |
| W_surface | 4.87±0.26 | 4.38±0.23 | 90.04±9.09 |
| W_outside | 5.11±0.25 | 4.68±0.21 | 91.57±8.61 |
| Y_0m | 1.33±0.14 | 0.76±0.05 | 57.07±9.76 |
| Y_5m | 1.02±0.07 | 0.75±0.06 | 73.51±10.95 |
| Y_surface | 4.42±0.47 | 3.6±0.28 | 81.45±15 |
| Y_outside | 5.86±0.28 | 5.08±0.20 | 86.69±7.54 |
